# Is the Renal Resistive Index a Marker for Revascularization in Atherosclerotic Renal Artery Stenosis?

**DOI:** 10.7759/cureus.14755

**Published:** 2021-04-29

**Authors:** Lalitha Padmanabha Vemireddy, Grace W Ying, Ammar Aqeel, Shaji Baig, Venkata Buddharaju

**Affiliations:** 1 Internal Medicine, Chicago Medical School Internal Medicine Residency Program, Northwestern McHenry Hospital, Chicago, USA; 2 Internal Medicine, Northwestern Medicine McHenry Hospital, Rosalind Franklin University of Medicine and Science, McHenry, USA; 3 Nephrology, Northwestern Medicine McHenry Hospital, Rosalind Franklin University of Medicine and Science, McHenry, USA

**Keywords:** atherosclerotic renal artery, renal artery stenosis, renal resistive index, renal artery stenting

## Abstract

Renal artery stenosis (RAS) is one of the major causes of resistant/malignant hypertension. It can be described as atherosclerotic or non-atherosclerotic. Atherosclerotic RAS comprises almost 90% of all RAS cases and is a prevalent disease of the elderly. Multiple risk factors contribute to atherosclerosis development, which leads to the release of renin and aldosterone, causing resistant/malignant hypertension. Early recognition is prudent but challenging as there are no early clinical signs. We believe that renal resistive index with supportive clinical, laboratory, and imaging modalities can help select revascularization patients.

## Introduction

Renal artery stenosis (RAS) is an important cause of malignant hypertension. Among the 50 million people in the United States diagnosed with hypertension, the incidence of malignant hypertension can be as high as 10% [1]. RAS is further categorized into atherosclerotic RAS (ARAS) and non-atherosclerotic RAS (N-ARAS) [2]. Atherosclerosis constitutes almost 90% of all RAS cases, with non-atherosclerotic causes making up the remaining portion [2]. With fibromuscular dysplasia being the most common cause of N-ARAS, some other contributing etiologies may include Takayasu’s arteritis, Buerger’s disease, thromboembolic disease, and infra-renal aneurysm [1,2]. Unlike N-ARAS, ARAS commonly occurs in the elderly and does have multiple risk factors such as hyperlipidemia, smoking, increased homocysteine levels, diabetes mellitus, family history, and old age [1,3,4]. Even though ARAS is only seen in 1%-6% of hypertension cases, it can present in as high as 50% of the elderly population with a known history of atherosclerotic disease [5]. ARAS treatment options usually consist of medical therapy, percutaneous angioplasty, or surgical intervention [2,4,5]. Management of RAS has been controversial as multiple studies have failed to establish one treatment therapy's superiority over another. We are presenting two cases of ARAS in patients with existing congestive heart failure (CHF) and discuss potential treatment challenges working with current management guidelines.

## Case presentation

Case 1

**Figure 1:**
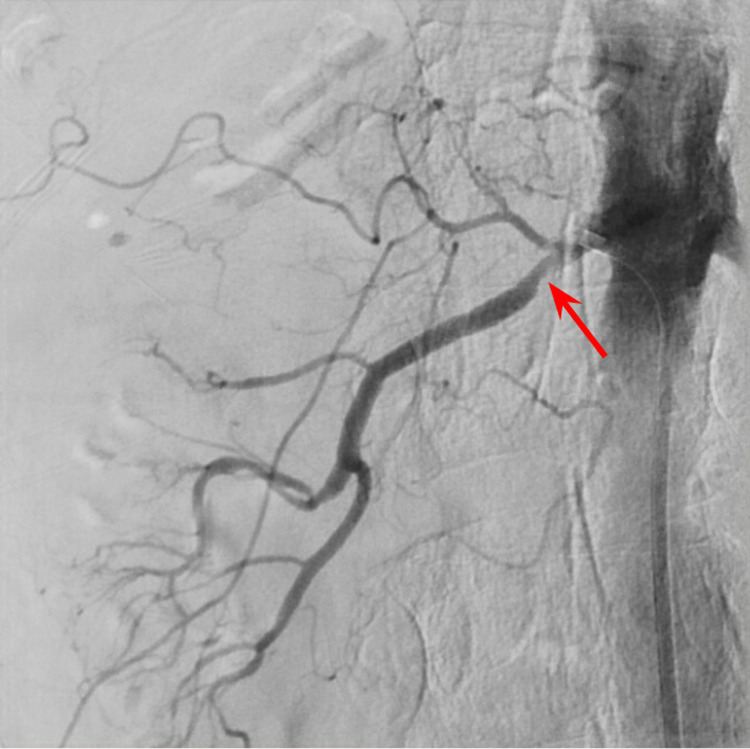
Right renal artery angiogram showing stenosis of the right renal artery (red arrow) close to the origin from descending aorta

**Figure 2:**
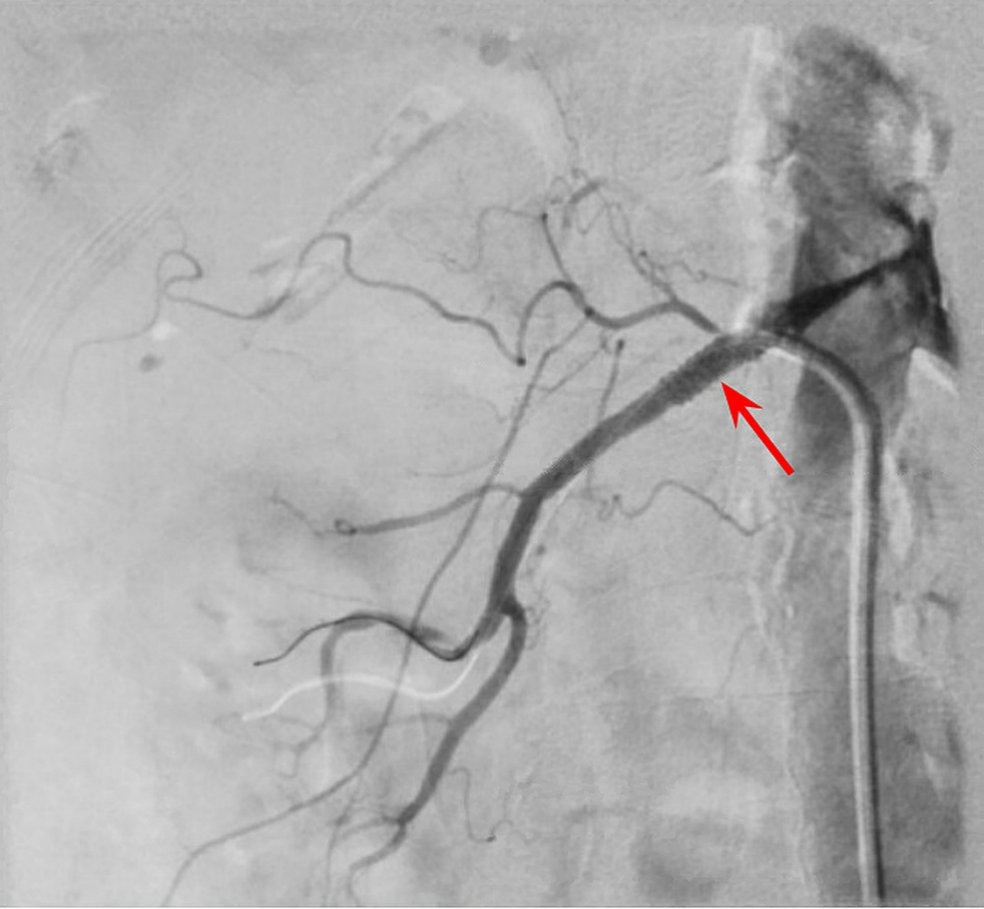
Right renal angiogram showing renal artery stent (red arrow) with no residual stenosis

An 88-year-old female with a past medical history of hypertension, coronary artery disease, combined systolic and diastolic CHF with a left ventricular ejection fraction of 40%, peripheral artery disease status post femoral bypass grafting, and diabetes mellitus type II, chronic kidney disease stage IIIB (calculated creatinine clearance of 31) presented to our hospital with complaints of dyspnea for 2-3-day duration. On presentation, the patient was found to be in a hypertensive emergency with blood pressure (BP) of 220/91 mmHg accompanied by signs of fluid overload. Lab studies were within normal limits. She was discharged after being treated with diuretics and anti-hypertensives. She presented again within a month with similar symptoms and BP of 188/79 mmHg. Due to recurrent flash pulmonary edema and resistant hypertension, a renal artery ultrasound was performed, which demonstrated atrophic right kidney and poorly visualized renal arteries. A subsequent renal angiogram showed 70% stenosis of the right renal artery, which was later intervened with a bare-metal stent (Figures 1, 2).

Nicardipine drip was initiated for worsening BP post-operatively in addition to the continuation of diuretics. As the patient's respiratory status worsened despite being on aggressive management, her family ultimately decided and opted for hospice. Unfortunately, our patient passed away a few hours later.

Case 2

**Figure 3:**
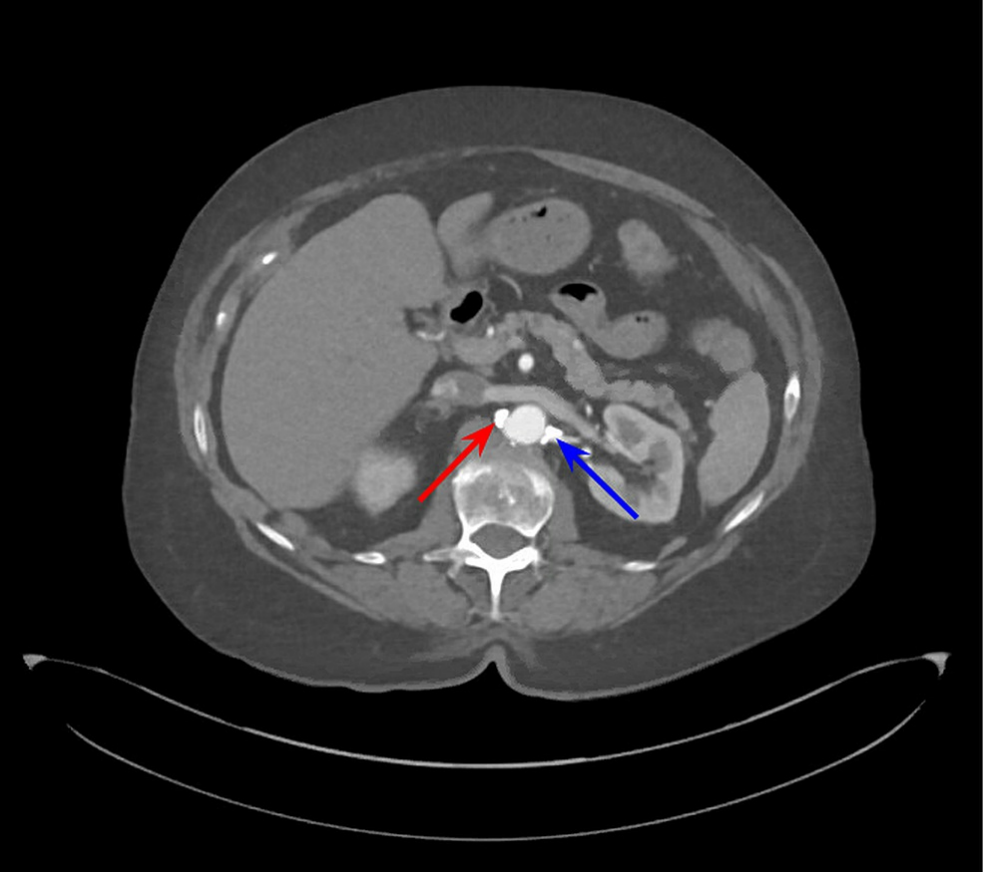
CTA abdomen demonstrating a high-grade stenosis of the right renal artery origin (red arrow) from aorta and >50% stenosis of the left renal artery (blue arrow) CTA - Computed tomographic angiography.

An 86-year-old female with a past medical history of hypertension, coronary artery disease status post percutaneous coronary intervention, diastolic CHF with a left ventricular ejection fraction of 69%, insulin-dependent type II diabetes mellitus, dyslipidemia, cerebrovascular disease, chronic kidney disease stage IIIB (calculated creatinine clearance of 39) and anxiety presented to the hospital with complaints of sub-sternal chest discomfort and frontal headache for several days. She had numerous admissions in the past for refractory hypertension. Upon being on multiple antihypertensive regimens, initial vital signs on this admission were stable except for a BP of 221/88 mmHg. Computed tomographic angiography of chest/abdomen/pelvis found high-grade stenosis at the right renal artery origin with at least 50% stenosis of the left renal artery origin (Figure 3).

**Figure 4:**
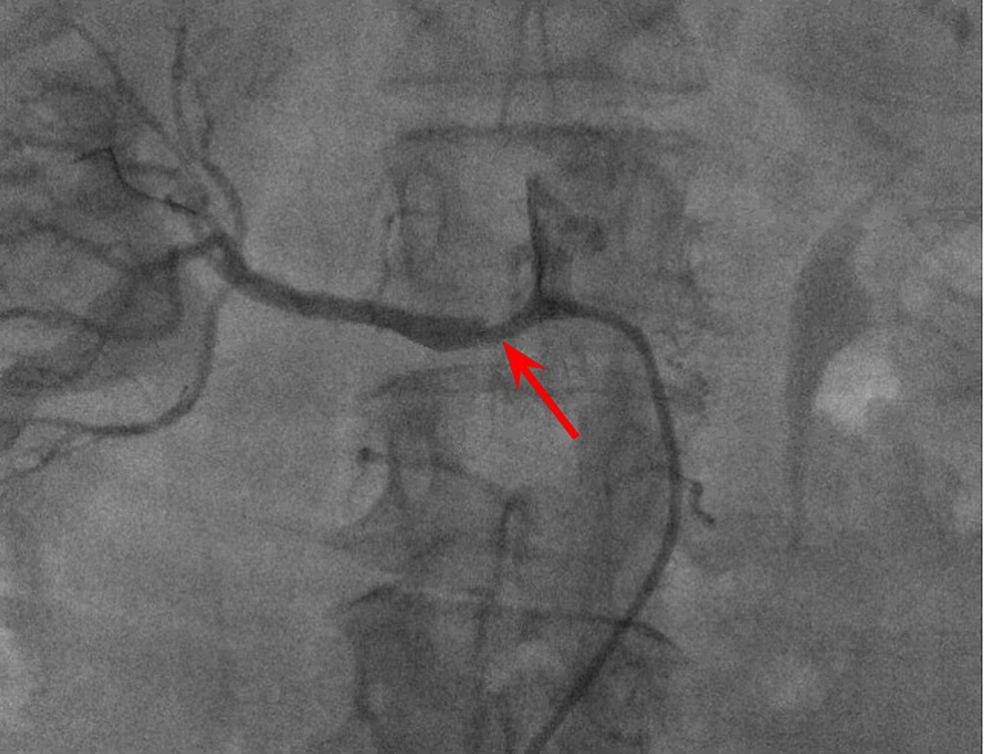
Right renal angiogram showing stenosis of the right renal artery (red arrow)

**Figure 5:**
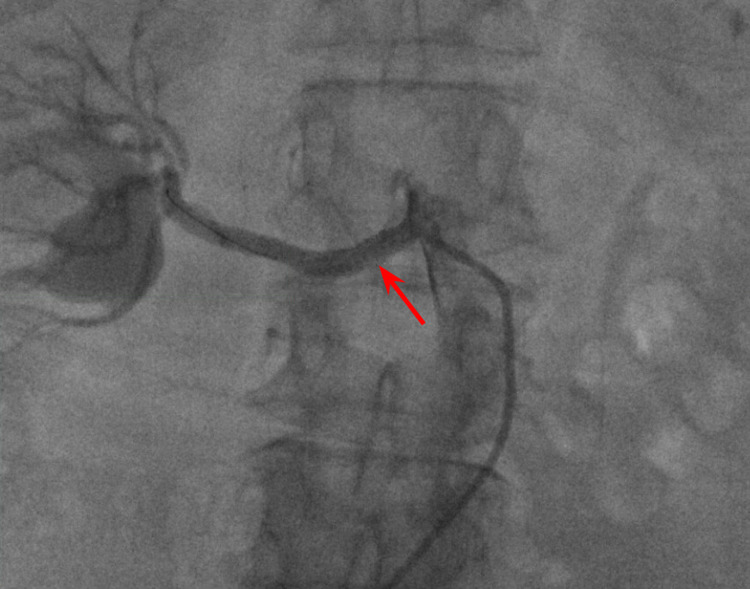
Right renal angiogram showing right renal artery with stent (red arrow) and no residual stenosis

She subsequently underwent renal angiography with a right renal artery stent placement and was discharged home on several antihypertensive medications (Figures 4, 5).

Her BP was not effectively lowered as expected following the procedure with the BP measured 198/80 mmHg during the outpatient cardiology office visit despite good medication adherence. The patient was readmitted to the hospital a week later for a hypertensive emergency with a BP of 180/70 mmHg and was discharged home again with numerous BP medications, including losartan, clonidine, and spironolactone.

## Discussion

As previously mentioned, ARAS constitutes almost 90% of RAS cases [2]. The primary mechanism of ARAS-induced hypertension is due to activation of the renin-angiotensin-aldosterone system leading to subsequent fluid overload. Any risk factor capable of causing endovascular injury can lead to accumulation of low-density lipoprotein, macrophages, and proliferation of endothelial and smooth muscle cells forming atherosclerotic plaques. Progressive plaque build-up results in a decrease in blood flow to the kidneys, which activates the juxtaglomerular apparatus (JGA). JGA subsequently releases renin, which produces angiotensin I. Angiotensin I is further converted into angiotensin II causing vasoconstriction and aldosterone production from the adrenal glands. Aldosterone contributes to salt and water retention leading to fluid overload and hypertension [1,2].

ARAS is often suspected with the presence of one or more clinical signs. These may include resistant hypertension (failure to achieve BP control with ≥3 antihypertensive agents of different classes), uncontrolled hypertension in patients >50 years of age, often with a history of carotid artery stenosis, coronary artery stenosis or peripheral arterial disease, azotemia, or worsening renal function especially after administration of angiotensin-converting enzyme inhibitors (ACEIs) or angiotensin receptor blockers (ARBs), unexplained hypokalemia, new-onset abdominal bruits, unexplained CHF, or recurrent flash pulmonary edema [2,5].

ARAS diagnosis can be made using Doppler ultrasonography, computed tomographic angiography, magnetic resonance angiography, invasive angiography, or renal scintigraphy scan (Captopril Renal Scan). Each of these imaging techniques comes with its own set of advantages and disadvantages. Ultimately the imaging of choice should be determined on a case-by-case basis considering varied comorbidities among patients [2,5,6].

When it comes to ARAS treatment, there are two different approaches: medical therapy and revascularization, including renal artery stenting and surgical revascularization. Medical therapy usually involves the initiation of antihypertensive agents, statins, risk factor modification like glycemic control and smoking cessation, and primary prevention with aspirin. The antihypertensive agents of choice are ACEI, ARBs, beta-blockers, and calcium channel blockers. While patients are on ACEI or ARBs, renal function should be monitored closely. On the other hand, renal artery revascularization can be achieved through balloon angioplasty with or without stenting or surgical revascularization. Several studies have shown that surgical revascularization is associated with higher rates of complication. Thus, percutaneous intervention is often preferred [2,5,7].

There are three major randomized clinical trials (STAR, ASTRAL, CORAL) comparing medical therapy with stent placement in patients diagnosed with ARAS. However, all of them have failed to show a statistically significant difference regarding mortality benefit, renal function, or BP control [8-10]. A retrospective study done by Kane et al. showed no statistical difference between the medical therapy group and the angioplasty group, with the long-term need for dialysis being equally high. However, there seemed to be some short-term improvement in renal function with angioplasty [11]. Radermacher et al. have been pivotal in measuring the renal resistive index (RRI) with Doppler ultrasound as a non-invasive measurement of renal hemodynamics. RRI, calculated as (peak systolic velocity - end-diastolic velocity)/peak systolic velocity, is associated with various cardiovascular events and mortality in elderly, hypertensive, and diabetic patients. As part of the screening process to determine the likelihood to benefit from intervention, Radermacher et al. came up with a list of criteria including age > 65, pulse pressure of at least 70 mmHg, CrCl < 40 mL/min, urine protein excretion of at least 1 gram per day, hyperuricemia, coronary artery disease, peripheral artery disease, and cerebrovascular disease [12].

Furthermore, Soulez et al. also proposed some valuable predictors of outcome after renal angioplasty. These include measurements of kidney length and unilateral and bilateral RRI before and after the administration of captopril. For example, bilateral renal artery RRI < 0.75 before captopril administration and unilateral RRI < 0.70 after captopril administration; or bilateral renal artery RRI < 0.75 before captopril administration and kidney length measuring longer than 90mm have been found to have favorable outcome [13]. A study done by Davies et al. analyzed renal parenchymal preservation and that patients who had parenchymal losses were found to have higher RRI. This was correlated with worse clinical outcomes and faster progression to dialysis [14]. Zeller et al. divided 176 patients into three groups based on RRI (RI < 0.7, RI 0.7-0.8, RI > 0.8), and it was found that the improvement in renal function and the reduction in BP were the same across all study groups [15].

Considering the factors Radermacher et al. proposed, our two patients met at least half of the criteria, so we believe that the intervention would not have changed the outcome in terms of BP control, renal function, or overall mortality. The current guidelines recommend an invasive strategy for those not responding well to optimal medical therapy or are subject to recurrent CHF exacerbations or flash pulmonary edema. Stricter criteria based on further clinical trials involving age, kidney function, diabetes, other comorbid conditions, laboratory data, RRI, etc. need to be developed. Based on these factors, suitable candidates should be selected who would benefit from revascularization.

## Conclusions

ARAS is a common cause of resistant/malignant hypertension especially in the elderly. Medical therapy still remains the mainstay of treatment while some patients require revascularization. As multiple randomized clinical trials have proven that revascularization does not improve mortality, renal function, or blood pressure, it is of utmost importance to develop a specific set of criteria including RRI and various clinical, laboratory, and imaging modalities to select patients who would benefit from revascularization.
